# Society Meetings

**Published:** 1911-08

**Authors:** 


### SOCIETY MEETINGS

---

The American Association of Obstetricians and Gynecologists will hold its twenty-fourth annual meeting at Louisville, Ky., September 26-28, 1911, under the presidency of Dr. Herman E. Hayd, of Buffalo. An interesting program is being prepared.

